# Laser-synthesized oxide-passivated bright Si quantum dots for bioimaging

**DOI:** 10.1038/srep24732

**Published:** 2016-04-22

**Authors:** M. B. Gongalsky, L. A. Osminkina, A. Pereira, A. A. Manankov, A. A. Fedorenko, A. N. Vasiliev, V. V. Solovyev, A. A. Kudryavtsev, M. Sentis, A. V. Kabashin, V. Yu. Timoshenko

**Affiliations:** 1 Lomonosov Moscow State University, Department of Physics, 119991 Moscow, Russia; 2 Bio-nanophotonics Laboratory, National Research Nuclear University “MEPhI” (Moscow Engineering Physics Institute), 31 Kashirskoe sh., 115409 Moscow, Russia; 3 Institut Lumière Matière, UMR5306 Université Lyon 1-CNRS, Université de Lyon, 69622 Villeurbanne cedex, France; 4 Institute of Theoretical and Experimental Biophysics, Russian Academy of Sciences, Pushchino, 142292, Moscow Region, Russia; 5 Aix Marseille University, CNRS, UMR 7341 CNRS, LP3, Campus de Luminy – case 917, 13288, Marseille Cedex 9, France

## Abstract

Crystalline silicon (Si) nanoparticles present an extremely promising object for bioimaging based on photoluminescence (PL) in the visible and near-infrared spectral regions, but their efficient PL emission in aqueous suspension is typically observed after wet chemistry procedures leading to residual toxicity issues. Here, we introduce ultrapure laser-synthesized Si-based quantum dots (QDs), which are water-dispersible and exhibit bright exciton PL in the window of relative tissue transparency near 800 nm. Based on the laser ablation of crystalline Si targets in gaseous helium, followed by ultrasound-assisted dispersion of the deposited films in physiological saline, the proposed method avoids any toxic by-products during the synthesis. We demonstrate efficient contrast of the Si QDs in living cells by following the exciton PL. We also show that the prepared QDs do not provoke any cytoxicity effects while penetrating into the cells and efficiently accumulating near the cell membrane and in the cytoplasm. Combined with the possibility of enabling parallel therapeutic channels, ultrapure laser-synthesized Si nanostructures present unique object for cancer theranostic applications.

The interaction of inorganic nanomaterials with biological systems has recently become a hot topic due to a variety of attractive applications in biomedicine. The interest to these materials is caused by a series of their unique properties (optical, photochemical, magnetic, electrical, mechanical etc.), which promise a drastic improvement of the current state-of-the-art imaging and therapeutic modalities^1^,^2^. In particular, some semiconductor-based nanostructures called quantum dots (QDs) can emit photoluminescence (PL) with much improved characteristics compared to organic fluorophores, including a wider absorption band, longer fluorescence lifetime, good photostability and spectral tuneability of the PL band etc., making them promising candidates for spectral multiplexing with a variety of biological imaging applications^3^,^4^. CdSe/CdS and some others compound QDs have been in the focus of many studies due to high quantum yield (QY) of their PL, which can exceed 50% for some structures^3^. QDs could be successfully conjugated to peptides^5^, proteins^6^ and DNA^7^ and tested in multi-color fluorescent imaging in cellular and animal models^8^,^9^. However, almost all conventional QDs cause critical toxicity problems. As an example, *CdSe-CdS* QDs provide extremely toxic *Cd*^*2*+^ ions under the interaction with biological environment. Although the short-term toxicity of QDs conjugates can somewhat be reduced by the proper polymeric or inorganic coating^10^, such nanostructure compounds are hardly accessible for clinical practice.

Silicon-based nanostructures present a viable alternative to compound QDs and they could offer a solution of the toxicity problem. Chemically pure silicon (*Si*) is a unique inorganic material, which is not only low toxic^11^, but also biodegradable as in biological environment it transforms into orthosilicic acid *Si(OH)*_*4*_, which is naturally excreted from the body with the urine^12^. Si nanostructures are also known as promising theranostic agents, which can simultaneously combine therapeutic and diagnostic (imaging) functionalities (theranostics = therapy + diagnostics). Indeed, from one side Si-based nanostructures can demonstrate efficient exciton PL with tunable emission band inside the human body transparency window (750–900 *nm*). In this case, a much longer emission lifetime (1–100 *μs*) of this PL band^13^ compared to tissue autofluorescence signals makes possible the development of imaging modalities based on time-gated suppression of noises^14^. On the other hand, Si nanostructures can be used as sensitizers for photodynamic therapy (PDT) to efficiently generate singlet form of molecular oxygen under photoexcitation and thus treat malignant tumors^15^,^16^,^17^. In addition, Si nanocrystals can sensitize hyperthermia, i.e. local heating of a tumor tissues above 42 °C leading to an efficient destruction of cancer cells, under their irradiation by infrared radiation^18^, ultrasound^19^ and radio-frequency waves^20^.

It is widely accepted that to obtain water-dispersible brightly luminescent *Si* QDs, one has to fabricate small (2–4 *nm*) *Si* nanocrystals with an appropriate passivation (coating) of their surface to remove non-radiative recombination centers^21^,^22^,^23^,^24^,^25^. The latter procedure usually requires a “hydrogenation” step by using solutions of hydrofluoric acid (*HF*) or a mixture of *HF* and nitric acids (*HNO*_*3*_), which inevitably leads to a surface contamination by acid derivatives drastically enhancing toxicity of the QDs (such toxicity arising as a result of the fabrication procedure can be called “secondary toxicity”). Here, a straightforward approach implies a mechanical milling of porous silicon, which is preliminarily produced on a crystalline *Si* (*c-Si*) wafer by anodic etching in HF/ethanol solutions and then oxidized by storage in air^16^,^18^ or ethanol^13^,^17^. Such a combination of hydrogenation and oxidation processes provides a hydroxyl-based passivation of the nanocrystals and the upper surface layer *SiO*_*y*_*H*_*x*_ (where *y* = 1…2*; x* = 0…2) ensures a good dispersion of QDs in aqueous solutions. In principle, brighly luminescent Si nanocrystals can be produced by much cleaner “dry” fabrication methods, including laser or plasma pyrolysis of silane^26^,^27^,^28^,^29^,^30^,^31^, thermal annealing of amorphous *SiO*_*x*_ films^23^,^24^,^27^, or laser ablation from a c-Si target^32^,^33^,^34^. However, the prospects of such nanocrystals as bioimaging markers are not clear, as the strong PL of solid nanostructured films does not necessarily guarantee a high PL efficiency of those nanocrystals after the dispersion in aqueous media. As an example, nanocrystals synthesized by the pyrolysis^22^,^26^,^27^ and thermal annealing of amorphous SiO_x_ films^23^,^24^,^27^ typically need to be subjected to a wet chemistry etching step in *HF* or *HF-HNO*_*3*_ solutions to spatially separate the nanocrystals, decrease their size, and condition the required hydroxyl-based passivation in order to obtain the desired PL emission. As a result, the secondary toxicity of QDs could not be avoided.

Here, we report on a solution of the secondary toxicity problem of brightly luminescent water-dispersible *Si*-based QDs by employing a laser-assisted synthesis. Formed by laser ablation from a solid *c-Si* target in gaseous *He* and grown in laser-plasma environment, *Si* nanoclusters experience nearly perfect crystallization, followed by surface passivation by oxygen during their exposure to air (oxide passivation). After the dispersion in aqueous solutions, the oxide-passivated *Si* QDs exhibit the strong exciton-based PL with QY of several percent without additional wet chemistry procedures. The QDs do not show any sign of toxicity, demonstrate biodegradability and excellent cellular uptake, which makes them ideal candidates for bioimaging applications.

## Results and Discussion

**Figure 1:**
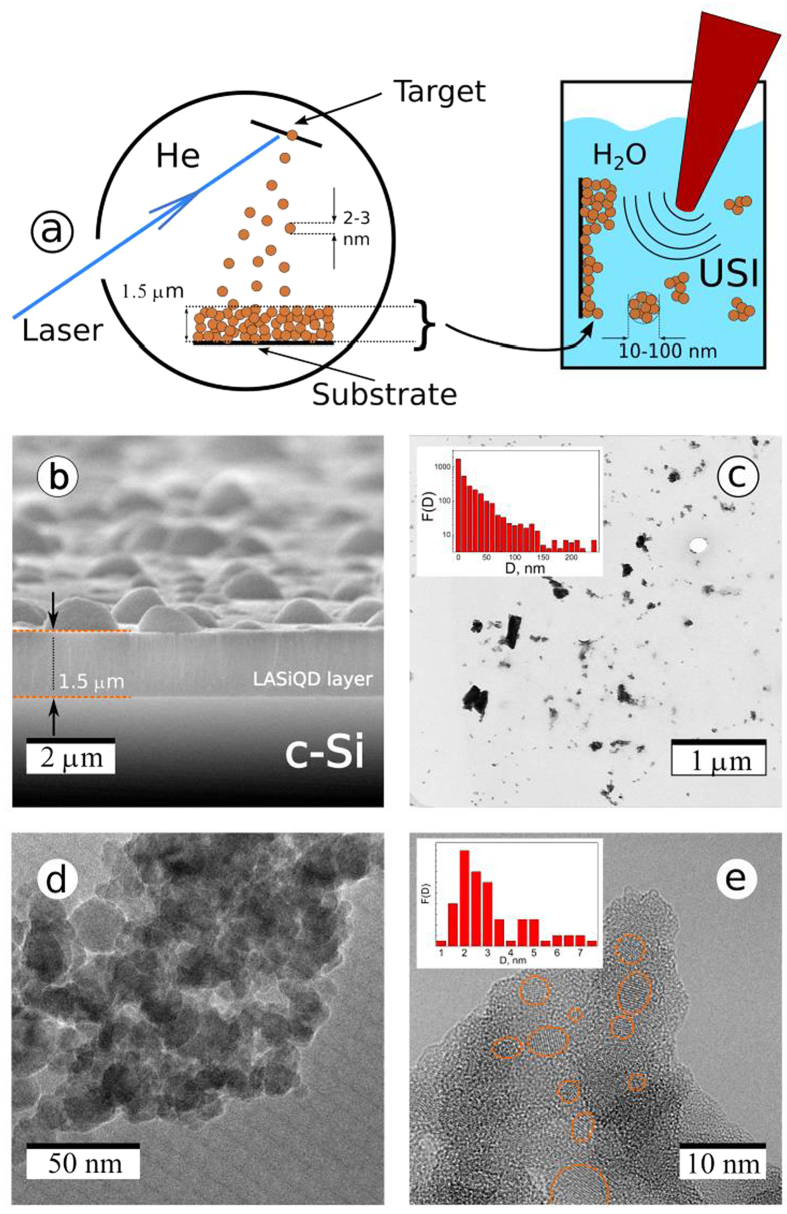
(**a**) Laser-ablative synthesis of Si nanostructures. (**a**) Schematics of two-step laser synthesis: Laser ablation of a *c-Si* target in residual *He* gas leads to the deposition of a nanostructured LA-*Si* film (left panel); the film is then treated by ultrasonic irradiation in an aqueous physiological solution resulting in the removal of *Si* nanocrystals and the formation of water-dispersed NPs; (**b**) Scanning electron Microscopy (SEM) image of laser-ablated *Si*-based nanostructured film deposited on *c-Si* substrate; Transmission Electron Microscopy (TEM) (**c**) and high resolution TEM images (**d,e**) of LA-*Si* NPs produced by ultrasound-based milling of laser-ablated nanostructured films. Orange ellipses depict *Si* nanocrystals.

The laser-ablative synthesis consists of two steps. As the first step, we use a technique of pulsed laser ablation of a solid *c-Si* target in gaseous ambience to deposit a thin nanostructured *Si*-based film on a substrate. Briefly, a *c-Si* wafer is irradiated by *UV* radiation of a *KrF* excimer laser in residual *He* gas (see Methods Section for details). The laser irradiation leads to ablation of material in the form of atoms and smallest nanoclusters, which move perpendicularly to the target surface, as shown schematically in Fig. 1a. *He* gas under the pressure of 1–5 *Torr* is used to finely control the growth and crystallization of nanoclusters: collisions of the nanoclusters with *He* atoms lead to their cooling, condensation and crystallization in the vapor phase^32^,^33^. The nanocrystals are then deposited on optically polished substrates of *c-Si* wafers, which are placed 2–3 *cm* from the target, to form a nanostructured film with the thickness of 1–5 *μm* (Fig. 1a). As shown in Fig. 1b, such films are highly porous with the estimated porosity of 65–75% (see details in Supplementary Information, Fig. S1). The films also contain hemispherical μm-size droplets due to the ejection of large target fragments, but these droplets do not affect the integrity and optical quality of the films. It is worth noting that the laser-ablated nanostructured Si-based films can be deposited on an arbitrary substrate (including a gold surface) and used in various biosensing configurations, including Surface Plasmon Resonance^35^,^36^. As the second stage, we perform an ultrasound-assisted breakage (sonification) of the laser-deposited films in deionized water or physiological saline (see Methods Section). The nanostructured porous films are detached from the substrate as a result of this sonification and dispersed in the solution forming a colloidal *Si* nanocrystal-based suspension.

**Figure 2:**
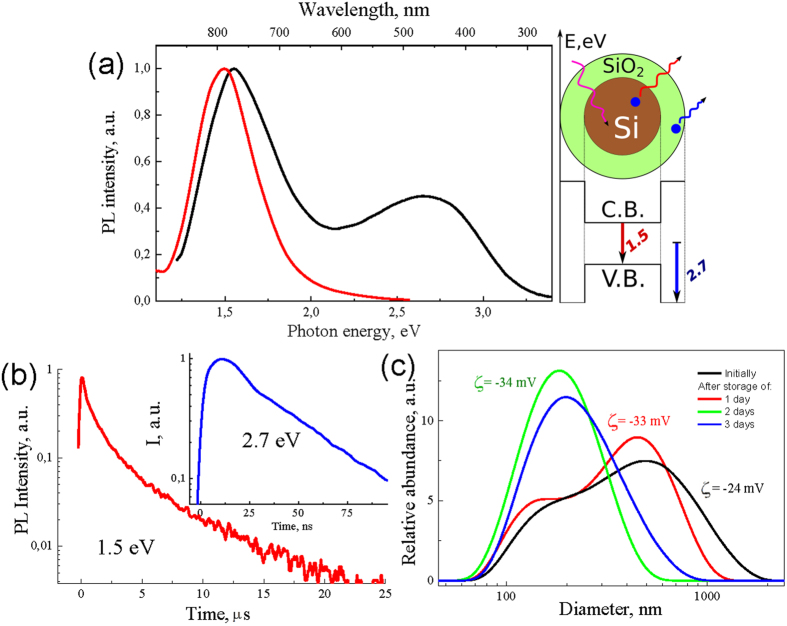
Properties of LA-*Si* QDs. **(a)** Photoluminesence spectra of LA-*Si* nanostructured films (red curve) and aqueous suspensions of LA-*Si* NPs (black curve); (**b**) typical PL transient for the exciton band (1.5 *eV*). The inset shows PL transient for the defect-related band at 2.7 *eV*; (**c**) Dynamic light scattering spectra from LA-*Si* QD agglomerates after different time of their storage in saline.

Figure 1c presents a typical transmission electron microscopy (TEM) image of *Si* nanoparticles (NPs) obtained after the sonification step and related size distribution of the NPs before any size filtering (inset). One can see that despite the presence of big agglomerates the majority of laser-ablated *Si* NPs (LA-*Si* NPs) have a diameter less than 100 *nm*, which makes them suitable for biomedical applications. Moreover, large LA-*Si* NPs present aggregations of small nanoparticles (Fig. 1d) and can be disintegrated during their storage in aqueous media (Fig. 2c). Fine structure of typical LA-*Si* NPs can be seen in the HRTEM image of Fig. 1e. It is visible that these LA-*Si* NPs are composed of randomly distributed crystalline grains (denoted by orange circles) incorporated into a porous matrix. Such a matrix is mostly composed of amorphous SiO_x_, as it was earlier evidenced^33^,^37^,^38^. As follows from the FTIR data (see Fig. S2 in Supplementary Information), the upper layer composition is close to silicon dioxide, i.e. *x* = 1.95 ± 0.05, but deeper layers evidence the presence of a fraction of the suboxide phase close to SiO_1.5_^38^. This could be interpreted as a non-uniform coating, implying nearly SiO_2_ compound at the surface and SiO_x_ (*x *< 2) for deeper layers, where *x* decreases with the increase of distance related to the crystalline Si core. According to NPs size distribution shown in the inset of Fig. 1e, the mean crystal size is 2.5 ± 0.5 *nm*, while QDs with sizes of 5–8 *nm* are also present. It should be noted that such small nanocrystal sizes are consistent with the manifestation of the quantum confinement effect in semiconductor nanostructures^13^.

The laser-ablated films exhibit strong PL signals just after their exposition to ambient air. As shown in Fig. 2a, the recorded PL band is spectrally centered at 1.5 *eV* (810 *nm*). While the band position is commonly explained by the quantum confinement of charge carriers in *Si* QDs^13^, the PL origin can be attributed to the radiative recombination of excitons either in the whole volume of QDs^13^,^39^ or on *Si-O* bonds at the *SiO*_*2*_*/Si* interface^40^. Note that the position of 1.5 eV exciton band corresponds to the average nanocrystal size 2.5 ± 0.5 nm, which is in good agreement with previous data on exciton PL of Si QDs embedded in a SiO_2_ matrix^41^. It is important that the exciton PL band does not disappear after the sonification of the LA-Si films and their subsequent dispersion in pure water or physiological saline (Fig. 2a). The storage of NPs in aqueous media leads to a slight blue-shift of the exciton PL band to 1.6 *eV*, which is probably explained by a partial oxidation of LA-*Si* NPs, and consequently, to a decrease of the size of pure Si core. The storage of LA-*Si* NPs in aqueous environment also leads to the appearance of the second PL band located at 2.7 *eV*, which is usually attributed to the electronic states of point defects in the *SiO*_*x*_ phase^41^,^42^. The most relevant candidates for the origin of blue band are neutral oxygen vacancies (≡Si-Si≡), which are molecular-like centers in silicon-rich or oxygen-deficient silicon oxides^43^,^44^. It is also important that the observed PL bands have quite different time decay dependences. Whereas the time decay of the defect-related band is very fast (tens of nanosecond for the 10-fold decrease of PL intensity), the relevant parameter for the exciton band is much slower (tens of μs), which is in agreement with previous studies of the exciton emission in porous silicon^13^ and nanostructured *Si*-based films^23^,^24^. The external QY of the spectrally integrated PL was found to be about 5% and 3% for the films and aqueous suspensions of LA-*Si* NPs, respectively. It should be noted that such QY values are orders of magnitude higher than in the case of *Si* NPs prepared by laser ablation in deionized water^45^,^46^.

The efficient PL from laser-ablated films and suspensions of *Si* QDs is a pleasant surprise, taking into account that *Si* nanostructures produced by most alternative dry methods such as laser pyrolysis of silane^1^,^22^,^26^ do not provide luminescence before an additional wet chemical treatment step in solutions of *HF-HNO*_*3*_. We believe that that the formation of strongly luminescent Si QDs in our case is due to particular conditions of crystal growth in laser-plasma plume and their subsequent passivation in ambient air. It is known that the plume of ablated *Si* nanoclusters is capable of efficiently ionizing atoms of ambient *He* gas and thus form plasma of a relatively long (>1 ms) lifetime^47^. Visible by a naked eye due to characteristic luminescence emission, such a region of the ionized gas plasma can reach the distances of 1.5–2 *cm* from the target. Although the nanoclusters move with a relatively high velocity (~10 m/s)^47^, they can get a significant amount of energy while propagating through the long living plasma before reaching the substrate. Such energy gain can be due to collisions of *Si* nanoclusters with *He* ions or hot electrons, which have a significant excess of energy of the order of tens *eV* in laser plasma^47^. It is known that even a single electron-ion recombination event in non-equilibrium plasma can increase the temperature of a small nanocluster up to 1000 *K*^48^. Therefore, we can expect a significant heating of laser-ablated *Si* nanoclusters during their propagation through the plasma of the ionized gas. We suppose that a subsequent cooling of the ultra-hot clusters under their collisions with cold *He* atoms (outside the plasma area) creates conditions for the formation of nearly-perfect low-defect *Si* QDs. Thus, the formation of high-quality nanocrystals requires the involvement of heating and cooling stages before the nanocrystals reach the substrate. As it was found in ref. 33, such conditions are possible under a relatively narrow range of *He* pressures. The optimal pressure depends on the laser energy and target-substrate distance, but for our experimental parameters it was typically in the range of 0.5–5 *Torr*. It is interesting that within this pressure range the nanocrystal size is almost independent of the pressure, while the film morphology is critically sensitive to this parameter. In particular, the increase of *He* pressure from 0.5 to 4–5 *Torr* leads to gradual increase of the porosity of the laser deposited films from 20% to 90%^33^. Here, for relatively high pressures of 3–4 *Torr* the substrate is covered by highly porous powder rather than films, suggesting an efficient crystallization of *Si* nanoclusters in the vapor phase before reaching the substrate. In our experiments pressures around 2 Torr were optimal from the point of view of maximization of PL signals. We believe that such a pressure provides optimal conditions for heating and cooling processes in the formation of Si QDs.

The oxygen–based passivation of the laser-ablated films is another important process to achieve a bright emission of *Si* QDs. In our experiments, the passivation of *Si* QDs by *Si-O* bonds takes place just under the exposition of the laser-ablated films to ambient air. As we showed in ref. 33, the PL intensity is further 5–10-fold enhanced during a subsequent storage of the crystals in dry air, which suggests an improved passivation due to the growth of SiO_x_ shell. Since the laser-ablated films and NPs consist of networks Si QDs (see Fig. 1d,e), their PL intensities should be dependent on the exciton migration between neighboring Si QDs. Here, the exciton migration can result in the decrease of total PL QY and such a process is probably controlled by non-radiative defects in large *Si* QDs and *SiO*_*x*_ shell^24^. On the other hand, the single exponential decay of the exciton PL intensity in the time scale above 1–5 μs after pulsed photoexcitation (see Fig. 2b) evidences a nearly perfect passivation of certain fraction of brightly luminescent *Si* QDs^23^. The surface passivation of laser-synthesized *Si* QDs by the *Si-O* bonds does not look very stable in aqueous ambience that is illustrated by the decrease of the PL QY and appearance of additional PL band around 2.7 *eV* (see Fig. 2a). This effect is probably related to a well-known dissolution property of *Si* NPs in aqueous media. However the dissolution time of LA-*Si* NPs having a native surface oxide in neutral *pH* solutions can last for several days^20^, which still makes possible efficient bioimaging studies.

**Figure 3:**
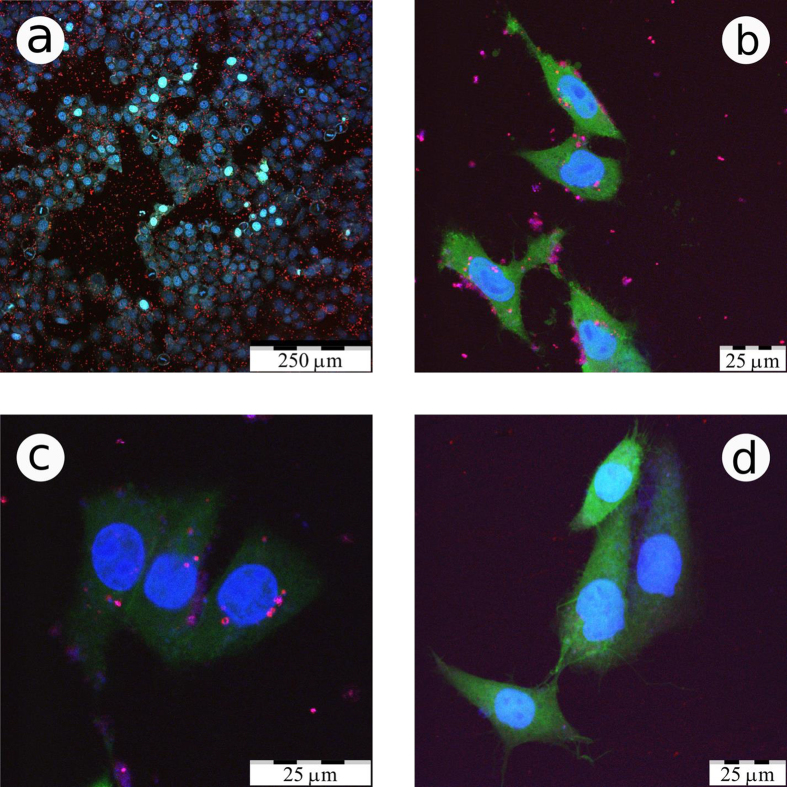
*In vitro* imaging of LA-*Si* NPs in cancer cells. Confocal fluorescence microscopy images of CF2Th cancer cells incubated with LA-*Si* NPs (colored red, pink and partially violet) under different magnification scales (**a–c**) and that of a control sample without NPs (**d**). Panel (**c**) presents the cells after washing out of LA-*Si* QDs from extracellular space. Cell nuclei are coloured blue, their cytoplasm is coloured green in panels (**b–d**).

To assess the potential of the prepared LA-*Si* NPs for biological imaging tasks, we carried out a series of tests on the incubation of nanoparticles in a cellular model. Figure 3 shows confocal fluorescent microscopy images of cancer cells with added LA-*Si* NPs under different magnifications. Cell nuclei are colored blue and the cytoplasm is colored green. Figure 3a shows the cells in different proliferation states, including mitotic cells in the metaphase. The last ones can be distinguished by metaphase plates, which look as blue rods substituting normal nuclei. These data evidence normal cell proliferation in the medium containing LA-*Si* NPs (red or red-blue spots) that confirms excellent biocompatibility of the prepared Si QDs. Figure 3b,c represent detailed views of cells before and after washing out of the nutrient solution with dispersed LA-*Si* NPs, correspondingly, and Fig. 3d represents a control group without NPs. It is visible that most LA-*Si* NPs emit both red and blue PL bands, which are in good agreement with their PL spectra in water (see Fig. 2b). In the images of Fig. 3 one can distinguish several groups of luminescent NPs: (i) free-floating NPs outside the cells; (ii) NPs stuck on the cell membrane; (iii) NPs penetrated into the cell cytoplasm; (iv) NPs concentrated near the cell nuclei. The observed variety of NPs locations evidences a non-selective mechanism of their penetration into the cells, i.e. endocytosis. Note that LA-Si NPs located near the nucleus membrane are hardly able to penetrate inside the nuclei, because the size of NPs is larger than the pore size of the nucleus membrane. The penetration of LA-*Si* NPs inside the cells is more easily seen in Z-scan imaging (see video file in Supplementary Information). The poor penetration of LA-*Si* NPs into the cell nuclei can be important to ensure their low genotoxicity similar to what was observed for NPs formed from porous *Si*^49^.

Thus, the incubation of *Si* QDs into living cells does not provoke any toxicity effects, while the NPs easily penetrate into the cells and concentrate in different cell regions except the nuclei. The presence of the NPs can be efficiently tracked by the red and blue emission of the PL bands of *Si* QDs.

## Conclusions

In conclusion, we prepared and investigated laser-ablated *Si* nanoparticles composed of small *Si* QDs as novel contrast agents for photoluminescent bioimaging. The nanoparticles were produced by methods of the pulsed laser ablation from a *c-Si* target in gaseous (*He*) ambience, followed by ultrasonic grinding of the laser-deposited films in aqueous solutions. By following the photoluminescence signals from *Si* QDs we evidenced the excellent uptake of *Si* nanoparticles by cancer cells and their efficient accumulation in different cellular regions. The employment of such QDs does not reveal any sign of residual cytotoxicity, making them extremely promising candidates for biological imaging tasks.

We believe that the laser plasma-assisted growth of small, low defect nanocrystals and their subsequent oxide passivation in ambient atmosphere is a very promising strategy for the creation of brightly luminescent, water-dispersible and non-toxic *Si* QDs. In the absence of wet chemistry step the *Si* QDs are exposed only to a clean environment (residual *He* gas, air, physiological solutions) and should have ultraclean surface. Although the photoluminescence QY of *Si* QDs (typically 3–5%) is slightly lower compared to *Si*-based nanostructures prepared by wet chemistry methods, it is sufficient to obtain efficient contrast in cellular or tissue imaging. In addition, the exciton emission appears to be exactly in the window of relative tissue transparency, which simplifies the implementation of imaging configurations. Another advantage is related to the μs-scale time decay of the PL signal from LA-*Si* NPs. Indeed, one can profit from such a long time decay to suppress much faster autofluorescence signals from biological molecules in the time-gated regime, as it was demonstrated *in vivo* in ref. 14. In fact, such lifetime multiplexing can increase signal-to-noise ratio by the factor of *k*, which is equal to contrast agent lifetime divided to the lifetime of autofluorescence (typically 1…50 *ns*). In our case the factor *k* can reach 1000. Finally, the prepared LA-*Si* NPs are water-soluble as it was revealed by a series of the DLS, TEM and Raman spectroscopy tests. As an example, the DLS analysis showed a six-fold decrease of the NPs size just after several days of nanoparticle storage in physiological solutions (Fig. 2c). Raman spectroscopy additionally revealed an increase of the amorphous *Si* phase up to 80% after 11 days of storage in aqueous suspension (see details in Supplementary Information, Fig. S3), which is obviously related to an efficient dissolution of Si NPs. This process excludes possible long-term toxicity (including genotoxicity) of the laser-ablated *Si* NPs. It should be noted that conditions of nanocrystal growth (plasma temperature and time scale of processes) in our case are similar in many respects to experiments on RF-decomposition of silane, which can also lead to the formation of brightly photoluminesent nanostructured *Si* films without any additional wet chemistry step^50^. Although we may not conclude on a complete similarity of these two cases, we can guess that that such RF radiation-formed Si nanocrystals can also be sonificated and water dispersed to obtain bright mobile QDs for bioimaging.

## Methods

At the first step, we used a conventional geometry of pulsed laser deposition in gaseous ambience. The radiation of a pulsed KrF laser (wavelength: 248 nm, pulse length: 17 ns, repetition rate: 10Hz) was used for the ablation of material from a rotating c-Si target ((100)-oriented *c-Si* wafer of n-type, specific resistance of 10 Ohm·cm)^33^. The radiation was focused on a focal spot of 2 mm^2^ on the target at the incident angle of 45° giving the radiation intensity of about 5*10^8^ W/cm^2^. c-Si wafer-based substrates, identical to the target, were placed on a rotating substrate holder at 2 *cm* from the target. The experimental chamber was pumped down to residual pressure of 10^−7^ *Torr* before filling with helium (purity 99.9995%) for a deposition at a constant pressure about 1–2 *Torr*. The film thickness after ten thousands laser shots was about 1 *μm*.

Aqueous suspensions of LA-*Si* NPs were obtained by ultrasonic treatment of the laser-ablated films in deionized water or saline (0.9% *NaCl* in *H*_*2*_*O*) for 1 *h*. The power density and frequency of ultrasound were 5 *W*/*cm*^2 ^and 44 *kHz*, respectively.

Scanning Electron Microscopy (SEM) images were obtained by using a Tescan Lyra 3 XM microscope with resolution of 1.2 *nm* and accelerating voltage of 30 *kV*. Transmission electron Microscopy (TEM) images were obtained by means of a Zeiss Libra 120 microscope with resolution of 0.5 *nm* and accelerating voltage of 120 *kV*. High resolution TEM images were provided by a JEOL JEM-2100F microscope with resolution of 0.8 *Å* and accelerating voltage of 200 *kV*. Size distributions of NPs and QDs were calculated by using ImageJ software.

PL spectra were measured by using a SOLAR spectrometer equipped with a CCD-unit from Hamamatsu. All spectra were corrected for the spectral response of the measurement systems. The PL transients were detected by a R928 photomultiplier tube (Hamamatsu Photonics, Hamamatsu, Shizuoka, Japan) under pulsed laser irradiation by a nitrogen laser (excitation wavelength of 337 *nm* and pulse duration of 10  *ns*). Time response of the detection system was shorter than 1 *μs*. PL quantum yield was measured by comparing the PL intensity and absorption of the samples with solutions of Rhodamine 6 G (PL QY about 100%).

To minimize noises in Raman measurements, we deposited a droplet of aqueous LA-Si NPs solution on a stainless steel samples. In addition, in a separate experiment we deposited nanostructured Si layers on CaF_2_ substrates. Raman spectra were measured by using a micro-Raman spectrometer from Horiba Jobin Yvon, excitation wavelength was 488 *nm*, maximum excitation power density was 10 *W/cm*^2^, spectral resolution was 0.1 *cm*^*−1*^. In order to avoid unfavorable heating a set of attenuating filters (0.3, 0.6, 1 and 2 dB) was used. Size distributions of NPs were measured by using a dynamic light scattering (DLS) Zetasizer ZS from Malvern. Porosity of LA-*Si* films and composition of LA-*Si* NPs were studied with a Fourier-transform infrared (FTIR) spectrometer Bruker IFS 66v/S. Before measuring the FTIR spectra, the suspensions of LA-*Si* NPs were deposited on an ATR crystal and then dried in air and evacuated at 10^−3 ^Torr.

*In vitro* bioimaging experiments were carried out with CF2Th (dog thymus) cells infected with a green fluorescent protein (GFP) gene. The latter was induced by RSL-1 inducer added to the CF2Th culture 30 h prior the bioimaging analysis. The GFP was characterized by a fluorescence line at 515 nm (green light). LA-*Si* NPs were introduced into the cell culture 5 *h* after the injection of the inducer and 25 *h* prior the experiment. 30 *min* prior the experiment, the cell nuclei were imbued with 5 *mg* Hoechst, which was luminescent near 460 *nm* (blue light). The cells containing LA-*Si* NPs were studied using a Leica TCS SP5 confocal fluorescent microscope with multicolor illumination at 488 nm, 496 nm, 514 nm, 543 nm and 633 nm to ensure simultaneous excitation of both the Si QDs and cells colored with GFP and stained with the Hoechst dye.

## Additional Information

**How to cite this article**: Gongalsky, M. B. *et al.* Laser-synthesized oxide-passivated bright Si quantum dots for bioimaging. *Sci. Rep.* **6**, 24732; doi: 10.1038/srep24732 (2016).

## Supplementary Material

Supplementary Information

## References

[b1] Prasad P. N. Introduction to Nanomedicine and Nanobioengineering. (J. Wiley & Sons Inc., New Jersey, 2012).

[b2] West J. L. & Halas N. J. Engineered Nanomaterials for Biophotonics Applications: Improving Sensing, Imaging, and Therapeutics. Annu. Rev. Biomed. Eng. 5, 285–292 (2003). 14527314 10.1146/annurev.bioeng.5.011303.120723

[b3] Michalet X. *et al.* Quantum Dots for Live Cells, *in Vivo* Imaging, and Diagnostics. Science 307, 538–544 (2005). 15681376 10.1126/science.1104274 PMC1201471

[b4] Medintz I. L., Uyeda H. T., Goldman E. R. & Mattoussi H. Quantum Dot Bioconjugates for Imaging, Labelling and Sensing. Nat. Mater. 4, 435–446 (2005). 15928695 10.1038/nmat1390

[b5] Whaley S. R., English D. S., Hu E. L., Barbara P. F. & Belcher A. M. Selection of Peptides with Semiconductor Binding Specificity for Directed Nanocrystal Assembly. Nature 405, 665–668 (2000). 10864319 10.1038/35015043

[b6] Bruchez M., Moronne M., Gin P., Weiss S. & Alivisatos a P. Semiconductor Nanocrystals as Fluorescent Biological Labels. Science 281, 2013–2016 (1998). 9748157 10.1126/science.281.5385.2013

[b7] Mitchell G. P., Mirkin C. a. & Letsinger R. L. Programmed Assembly of DNA Functionalized Quantum Dots [10]. J. Am. Chem. Soc. 121, 8122–8123 (1999).

[b8] Akerman M. E., Chan W. C. W., Laakkonen P., Bhatia S. N. & Ruoslahti E. Nanocrystal Targeting *in Vivo*. Proc. Nat. Acad. Sci. USA 99, 12617–12621 (2002). 12235356 10.1073/pnas.152463399 PMC130509

[b9] Gao X., Cui Y., Levenson R. M., Chung L. W. K. & Nie S. *In Vivo* Cancer Targeting and Imaging with Semiconductor Quantum Dots. Nat. Biotechnol. 22, 969–976 (2004). 15258594 10.1038/nbt994

[b10] Derfus A. M., Chan W. C. W. & Bhatia S. N. Probing the Cytotoxicity of Semiconductor Quantum Dots. Nano Lett. 4, 11–18 (2004). 10.1021/nl0347334 PMC5588688 28890669

[b11] Canham L. T. Bioactive Silicon Structure Fabrication through Nanoetching Techniques. Adv. Mater. 7, 1033–1037 (1995).

[b12] Park J.-H. *et al.* Biodegradable Luminescent Porous Silicon Nanoparticles for *in Vivo* Applications. Nat. Mater. 8, 331–336 (2009). 19234444 10.1038/nmat2398 PMC3058936

[b13] Kovalev D., Heckler H., Polisski G. & Koch F. Optical Properties of Si Nanocrystals. Phys. Stat. Sol. 215, 871–932 (1999).

[b14] Gu L. *et al.* *In Vivo* Time-Gated Fluorescence Imaging with Biodegradable Luminescent Porous Silicon Nanoparticles. Nat. Commun. 4, 2326 (2013). 23933660 10.1038/ncomms3326 PMC4154512

[b15] Timoshenko *et al.* Silicon nanocrystals as photosensitizers of active oxygen for biomedical applications. JETP Lett. 83, 423–426 (2006).

[b16] Xiao L., Gu L., Howell S. B. & Sailor M. J. Porous silicon nanoparticles photosensitizers for singlet oxygen and their phototoxicity against cancer cells. ACS Nano 5, 3651–3659 (2011). 21452822 10.1021/nn1035262 PMC3104024

[b17] Rioux D. *et al.* Silicon Nanoparticles Produced by Femtosecond Laser Ablation in Water as Novel Contamination-Free Photosensitizers”. J. Biomed. Optics 14, 021010 (2009) 10.1117/1.3086608 19405723

[b18] Lee C. *et al.* Porous Silicon as an Agent for Cancer Thermotherapy Based on near-Infrared Light Irradiation. J. Mater. Chem. 18, 4790–4795 (2008).

[b19] Sviridov A. P. *et al.* Porous Silicon Nanoparticles as Sensitizers for Ultrasonic Hyperthermia. Appl. Phys. Lett. 103, 193110 (2013).

[b20] Tamarov K. P. *et al.* Radio Frequency Radiation-Induced Hyperthermia Using Si Nanoparticle-Based Sensitizers for Mild Cancer Therapy. Sci. Rep. 4, 7034 (2014). 25391603 10.1038/srep07034 PMC5382688

[b21] English D. S., Pell L. E., Yu Z., Barbara P. F. & Korgel B. A. Size Tunable Visible Luminescence from Individual Organic Monolayer Stabilized Silicon Nanocrystal Quantum Dots. Nano Lett. 2, 681–685 (2002).

[b22] Erogbogbo F. *et al.* Biocompatible Luminescent Silicon Quantum Dots for Imaging of Cancer Cells. ACS Nano 2, 873–878 (2008). 19206483 10.1021/nn700319z PMC2676166

[b23] Sugimoto H., Fujii M., Imakita K., Hayashi S. & Akamatsu K. Codoping N- and P-Type Impurities in Colloidal Silicon Nanocrystals: Controlling Luminescence Energy from below Bulk Band Gap to Visible Range. J. Phys. Chem. C 117, 11850–11857 (2013).

[b24] Sangghaleh F., Sychugov I., Yang Z., Veinot J. G. C. & Linnros J. Near-Unity Internal Quantum Efficiency of Luminescent Silicon Nanocrystals with Ligand Passivation. ACS Nano 9, 7097–7104 (2015). 26083194 10.1021/acsnano.5b01717

[b25] Li Z. F. & Ruckenstein E. Water-Soluble Poly(acrylic Acid) Grafted Luminescent Silicon Nanoparticles and Their Use as Fluorescent Biological Staining Labels. Nano Lett. 4, 1463–1467 (2004).

[b26] Li X., He Y. & Swihart M. T. Surface Functionalization of Silicon Nanoparticles Produced by Laser-Driven Pyrolysis of Silane followed by HF-HNO_3_ Etching. Langmuir 20, 4720–4727 (2004). 15969188 10.1021/la036219j

[b27] Pavesi L. & Tura R. Silicon Nanocrystals: Fundamentals, Synthesis and Applications. (Wiley-VCH, Weinheim, 2010).

[b28] Anthony R., Rowe D., Stein M., Yang J. & Kortshagen U. Routes to achieving high quantum yield luminescence from gas-phase-produced silicon nanocrystals. Adv. Func. Mat. 21, 4044–4046 (2011).

[b29] Ledoux G. *et al.* Photoluminescence properties of silicon nanocrystals as a function of their size. Phys. Rev. B 62, 15942–15951 (2000).

[b30] Pi X. D. *et al.* Air-stable full-visible-spectrum emission from silicon nanocrystals synthesized by an all-gas-phase plasma approach. Nanotechnology 19, 245603 (2008). 21825815 10.1088/0957-4484/19/24/245603

[b31] Sankaran R. M., Holunga D., Flagan R. C. & Giapis K. P. Synthesis of Blue Luminescent Si Nanoparticles Using Atmospheric-Pressure Microdischarges. *Nanolett*. 5, 537 (2005). 10.1021/nl0480060 15755110

[b32] Kabashin A. V. *et al.* Nanofabrication with Pulsed Lasers Nanoscale Res. Lett. 5, 454–463 (2010). 20672069 10.1007/s11671-010-9543-z PMC2894200

[b33] Kabashin A. V., Sylvestre J., Patskovsky S. & Meunier M. Correlation between Photoluminescence Properties and Morphology of Laser-Ablated SiO/SiO_x_ Nanostructured Films. J. Appl. Phys. 91, 3248–3254 (2002).

[b34] Kabashin A. V. & Meunier M. Laser-induced treatment of silicon in air and formation of Si/SiO_x_ photoluminescent nanostructured layers, Mat. Sci. Eng. B 101, 60–64 (2003).

[b35] Patskovsky S., Bah S., Meunier M. & Kabashin A. V. Characterization of high-refractive index semiconductor films by Si-based Surface Plasmon Resonance”. Appl. Opt. 45, 6640–6645 (2006). 16912808 10.1364/ao.45.006640

[b36] Patskovsky S., Kabashin A. V., Meunier M. & Luong J. H. T. Si-based surface plasmon resonance sensing with two surface plasmon polariton modes. Appl. Opt. 42, 6905 (2003). 14661802 10.1364/ao.42.006905

[b37] Nakamura M., Mochizuki Y., Usami K., Itoh Y. & Nozaki T. Infrared absorption spectra and compositions of evaporated silicon oxides (SiO_x_). Solid State Comm. 50, 1079–1081 (1984).

[b38] Kabashin A. V., Meunier M. & Leonelli R. Photoluminescence Characterization of Si-Based Nanostructured Films Produced by Pulsed Laser Ablation. J. Vac. Sci. Technol. B 19, 2217 (2001).

[b39] Delerue C., Lannoo M. & Allan G. Excitonic and Quasiparticle Gaps in Si Nanocrystals. Phys. Rev. Lett. 84, 2457–2460 (2000). 11018909 10.1103/PhysRevLett.84.2457

[b40] Wolkin M. V., Jorne J., Fauchet P. M., Allan G. & Delerue C. Electronic States and Luminescence in Porous Silicon Quantum Dots: The Role of Oxygen. Phys. Rev. Lett. 82, 197–200 (1999).

[b41] Shalygina O. A. *et al.* Optical Properties of Silicon Nanocrystals in Silicon Dioxide Matrix Over Wide Ranges of Excitation Intensity and Energy. J. Nanoelectr. & Optoelectr. 4, 147–151 (2009).

[b42] Fitting H.-J. *et al.* Cathodoluminescence of Ge+, Si+, and O+ Implanted SiO_2_ Layers and the Role of Mobile Oxygen in Defect Transformations. J. Non. Cryst. Solids 303, 218–231 (2002).

[b43] Nishikawa H. *et al.* Photoluminescence from defect centers in high-purity silica glasses observed under 7.9-eV excitation. Phys. Rev. B 45, 586–591 (1992). 10.1103/physrevb.45.586 10001096

[b44] Rebohle L., von Borany J., Frob H. & Skorupa W. Blue photo- and electroluminescence of silicon dioxide layers ion-implanted with group IV elements. Appl. Phys. B 71, 131–151 (2000).

[b45] Svrcek V., Mariotti D. & Kondo M.. Ambient-stable blue luminescent silicon nanocrystals prepared by nanosecond-pulsed laser ablation in water. Opt. Express 17, 520 (2009). 19158863 10.1364/oe.17.000520

[b46] Blandin P. *et al.* Femtosecond laser fragmentation from water-dispersed microcolloids: toward fast controllable growth of ultrapure Si-based nanomaterials for biological applications. J. Mater. Chem. B, 1, 2489–2495 (2013). 10.1039/c3tb20285b 32261049

[b47] Geohegan D. B., Puretzky A. A., Duscher G. & Pennycook S. J. Time-resolved imaging of gas phase nanoparticle synthesis by laser ablation. Appl. Phys. Lett. 72, 2987–2989 (1998).

[b48] Mangolini L. & Kortshagen U. Selective nanoparticle heating: another form of nonequilibrium in dusty plasmas. Phys. Rev. E 79, 026405 (2009). 10.1103/PhysRevE.79.026405 19391853

[b49] Durnev A. D. *et al.* Evaluation of Genotoxicity and Reproductive Toxicity of Silicon Nanocrystals. Bull. Exper. Biol. & Medicine 149, 445–449 (2010). 10.1007/s10517-010-0967-3 21234440

[b50] Mangolini L., Thimsen E. & Kortshagen U. High-yield plasma synthesis of luminescent silicon nanocrystals. Nano Lett. 5, 655–659 (2005). 15826104 10.1021/nl050066y

