# Supplementary material for: Laser-synthesized oxide-passivated bright Si quantum dots for bioimaging
Source: Sci Rep. 2016 Apr 22;6:24732. doi: 10.1038/srep24732 (PMC4840388; doi:10.1038/srep24732)
Supplement: Supplementary Information [file srep24732-s1.pdf]

Supplementary Information to

Laser-synthesized oxide-passivated bright Si quantum dots for bioimaging

M. B. Gongalsky[1], L.A. Osminkina[1,2], A. Pereira[3], A. A. Manankov[1], A. A. Fedorenko[1], A. N. Vasiliev[1], V. V. Solovyev[4], A. A. Kudryavtsev[4], M. Sentis[2,5], A. V. Kabashin[5], V. Yu. Timoshenko[1,2]

[1]Lomonosov Moscow State University, Department of Physics, 119991 Moscow, Russia
[2] Bio-nanophotonics Laboratory, National Research Nuclear University “MEPhI” (Moscow Engineering Physics Institute), 31 Kashirskoe sh., 115409 Moscow, Russia
[3]Institut Lumière Matière, UMR5306 CNRS, Université Lyon 1, 10 rue Ada Byron, 69622 Villeurbanne, France
[4]Institute of Theoretical and Experimental Biophysics, Russian Academy of Sciences, Pushchino, 142292, Moscow Region, Russia
[5]Aix Marseille University, CNRS, UMR 7341 CNRS, LP3, Campus de Luminy – case 917, 13288, Marseille Cedex 9, France

1. FTIR analyses of the porosity of laser-ablated films

Fourier transform infra-red (FTIR) spectroscopy in the near- and middle IR ranges was used to determine both the effective refraction index, *n*, and width, *d*, of the obtained LA-Si films. The films were deposited on GaF_2 substrates, which were transparent in the investigated spectral region. Typical reflection spectra measured at different angles of incident are shown in Fig.1. The spectra consist of oscillations of the Fabry-Perot interference with maxima described by the following equation:

$$2kd\sqrt{n^2 - sin^2\alpha} = m, \quad (1)$$

where *k* is the wavenumber, *α* is the angle of incidence, *m* is the number of interference order.

Taking into account the same number maxima at different angles of incidence, one can estimate both *n* and *d*.

The value of *n* was used to estimate porosity, *P*, of the samples by using an effective medium approximation (EMA) based on Bruggeman approximation[1]:

$$f_1 \frac{\varepsilon_1 - \varepsilon_{eff}}{\varepsilon_1 + 2\varepsilon_{eff}} + f_2 \frac{\varepsilon_2 - \varepsilon_{eff}}{\varepsilon_2 + 2\varepsilon_{eff}} = 0, \quad (2)$$

where ε_{eff} is the effective dielectric function ($\varepsilon_{eff} = n^2$); ε_1 and ε_2 are the dielectric permittivities of Si nanocrystals and air, respectively; f_1 and f_2 are the corresponding filling factors ($P \equiv f_2$=$1 - f_1$).

The reflection data of Fig.1S were analyzed by using Eqs.1 and 2 to estimate the porosity of LA-Si films, which accounted P=0.70±0.05.

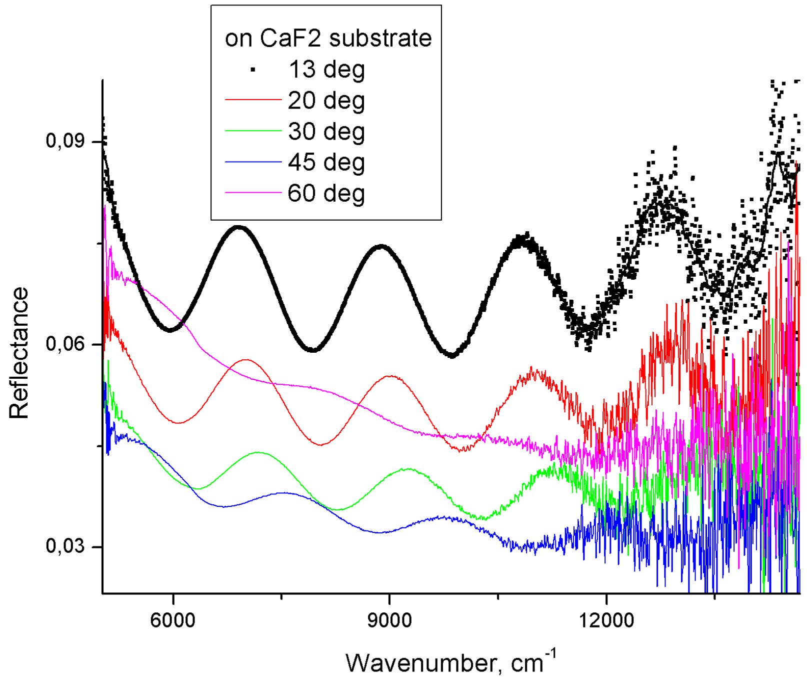

Fig. S1. FTIR reflectance spectra of a LA-Si film deposited on CaF_2 substrate and measured at different angles of incidence.

Note that more accurate evaluation of P should take into account SiO_x surrounding of Si nanocrystals as the third component of the effective medium[2]. Nevertheless, the porosity value of P=70% agrees well with the data obtained by using specular x-ray reflectivity for similar laser-ablated layers produced at 2 Torr of He[3].

2. FTIR analyses of the composition of LA-Si NPs

The chemical composition of LA-Si NPs deposited from the aqueous suspension on an ATR crystal and evacuated at 10^{-3} Torr was analyzed by means of FTIR spectroscopy. Fig. S2 shows transmittance spectrum of LA-Si NPs after 4 days of storage in aqueous medium. The observed absorption peak between 1000 and 1200 cm^{-1} corresponds to the Si-O valence vibrations.[4] The stoichiometry parameter, x, can be estimated from the absorption peak position as it is described in Ref. 5: it should be equal to 1082 cm^{-1} for SiO_2 and 980 cm^{-1} for SiO. One can obtain x = 1.95±0.05 for the spectral peak position 1080 cm^{-1} shown in Fig. 2S, evidencing nearly perfect dioxide composition of the NP surrounding. It is important that the FTIR spectrum in Fig. 2S contains an additional peak at 875 cm^{-1}, which can be attributed to SiO_x layer with x=1.55. As this peak vanishes for both x=1 and x=2, one can conclude that the suboxide layer is non-uniform and its stoichiometry may vary while going to deeper layers. Nevertheless, the $SiO_{1.5}$ phase is not dominating as it is accompanied by a Si-O valence peak shifted to 1040 cm^{-1}. The latter peak can be interpreted as a shoulder of the main peak at 1077 cm^{-1}.

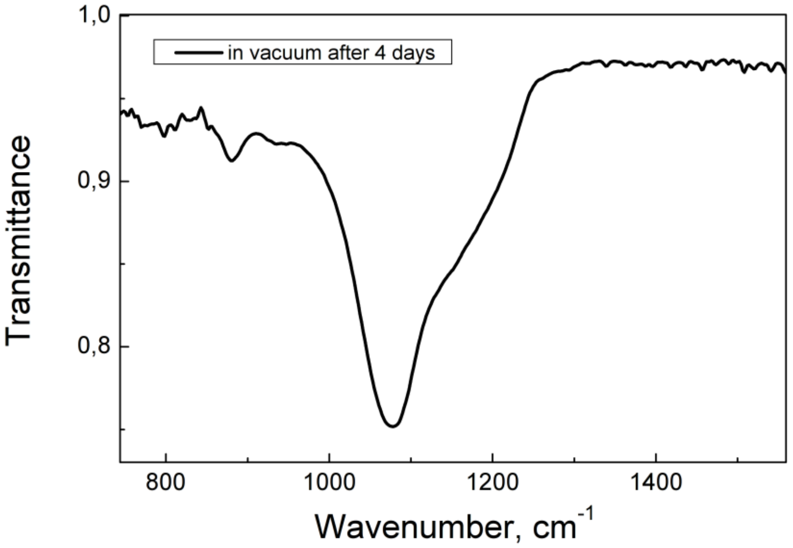

Fig. S2. FTIR spectra of LA-Si NPs after 4 days of storage in aqueous medium.

3. Raman spectroscopy of LA-Si NPs

LA-Si NPs deposited from aqueous suspensions on metal (stainless still) and initial LA-Si NPs layers deposited on CaF_2 substrates were investigated by using the Raman spectroscopy to estimate the mean size of Si QDs. Raman spectrum of the samples is shown in Fig. 3S. Here, a narrow peak near 520 cm^{-1} corresponds to the nanocrystalline *Si* phase, whereas a broad band centered at 480 cm^{-1} corresponds to the amorphous Si phase[6]. Fig.3S shows that the phase composition of the samples changes dramatically during storage in aqueous medium. It can be explained by dissolution of LA-Si QDs in water that is accompanied with strong disordering of the crystalline lattice of smallest Si QDs, i.e. their transformation to the amorphous ones. The low-frequency shift, $\Delta\omega$, of the Si nanocrystal peak is explained by size decrease of LA-Si QDs due to the phonon confinement[7]. To estimate the mean size, *D*, of Si QDs we use the following formula[8]:

$$\Delta\omega = -52.3\left(\frac{0.543}{D}\right)^{1.586}. \qquad (3S)$$

The measured peak position (Fig.3S) indicates that the mean size of QDs reduced from 15 to 5 nm after 11 days of storage in water. The size decrease can be explained by dissolution of Si NPs in water.

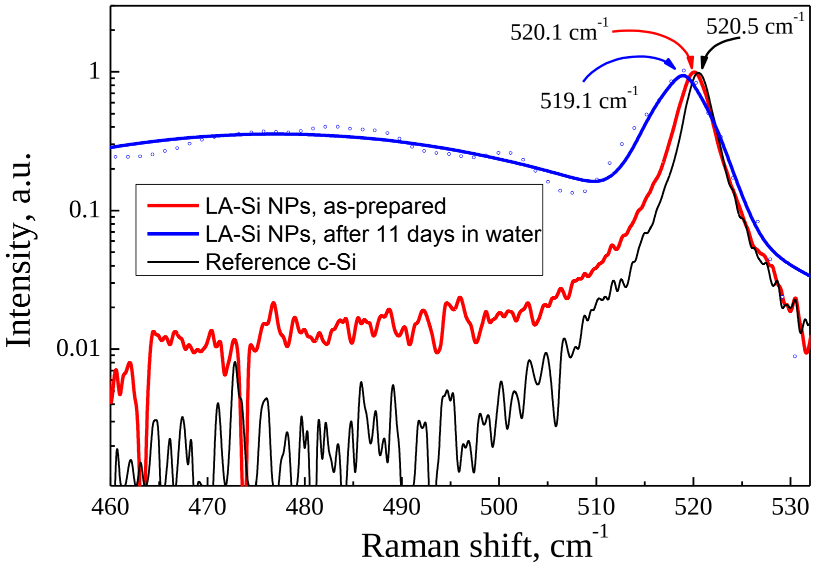

Fig. S3. Raman spectra of as-prepared LA-Si NPs (red curve) and those after 11 days of storage in water (blue curve). Black curve represents the reference c-Si spectrum. Arrows point main peak position.

4. Z-scan imaging of cancer cells with LA-Si NPs

Spatial localization of LA-*Si* NPs in cancer cells is illustrated by images of the confocal fluorescent microscopy with Z-step of 0.29 µm (z_scan_cells_Si_NPs.avi).

References:

1. Bruggeman, D. A. G. Dielectric constants and conductivity mixtures of isotropic materials. *Ann. Phys.* **24**, 636 (1935).
2. Astrova, E. V.; Tolmachev, V. A. Effective refractive index and composition of oxidized porous silicon films. *Mat. Sci. & Eng*. B **69**, 142-148 (2000).
3. Kabashin, A. V.; Sylvestre, J.; Patskovsky, S.; Meunier, M. Correlation between Photoluminescence Properties and Morphology of Laser-Ablated SiO/SiO_x Nanostructured Films. J. Appl. Phys. **91**, 3248–3254 (2002).
4. Thiess, W. Optical properties of porous silicon. *Surf. Sci. Rep.* **29**, 91–192 (1997).
5. Nakamura, M.; Mochizuki, Y.; Usami, K.; Itoh, Y.; Nozaki, T. Infrared absorption spectra and compositions of evaporated silicon oxides (SiO_x). *Solid State Comm*. **50**, 1079-1081 (1984).
6. Maley, N.; Beeman, D.; Lannin, J. S. Dynamics of tetrahedral networks: Amorphous Si and Ge. *Phys. Rev. B 38*, 10611 (1988).

7. Campbell, I.H., Fauchet, P.M. The effects of microcrystal size and shape on the one phonon Raman spectra of crystalline semiconductors. *Solid State Commun.* **58**, 739 (1986).
8. Zi, J.; Zhang, K.; Xie, X. Comparison of models for Raman spectra of Si nanocrystals. *Phys. Rev. B* **55**, 9263 (1997).
